# Iliotibial band release as an adjunct to the surgical management of patellar stress fracture in the athlete: a case report and review of the literature

**DOI:** 10.1186/1758-2555-1-15

**Published:** 2009-07-30

**Authors:** Anthony Keeley, Paul Bloomfield, Peter Cairns, Robert Molnar

**Affiliations:** 1 Sydney Orthopaedic Trauma and Reconstructive Surgery, Sydney, Australia; 2 Narrabeen Sports Medicine Centre, Sydney Academy of Sport, Sydney, Australia

## Abstract

Stress fracture of the patella is rare. In this report, a case of patellar stress fracture occurring in an amateur athlete is presented, and an operative adjunct to the surgical management of this condition is proposed.

A review of the English literature identified 21 previous cases of stress fracture of the patella, the majority in young athletes. None of these reports discussed treatment addressing the pathological process contributing to patellar stress fracture.

The subject of this case report is a young male netballer who presented with a transverse stress fracture in the inferior third of his patella, on a background of patellofemoral overload. The patient underwent open reduction and internal fixation of his patella, combined with release of the iliotibial band. He returned to training after 6 weeks.

The previous literature suggests that operative fixation is indicated for the treatment of displaced patellar stress fractures. Iliotibial band release, as a surgical adjunct to this treatment, may address the pathology of these fractures, and facilitate a return to sport at the highest level.

## Background

Stress fracture of the patella is rare, with only 21 cases previously reported in the English literature.[1-13] Patellofemoral contact stress, combined with tension from the extensor mechanism, leads to increased tensile stress on the anterior surface of the patella with subsequent microfracture and propagation. Undisplaced fractures are usually managed non-operatively, while displaced fractures are best treated operatively. In most cases patients report a return to sport at close to premorbid levels usually between 3 and 6 months. No previous reports discuss treatment addressing the pathological process contributing to patellar stress fracture.

We report a case of displaced transverse stress fracture of the patella in an amateur athlete. In addition to operative fixation of the fracture, the patient also underwent surgical release of his iliotibial band (ITB). This procedure attempts to decrease patellofemoral stress and may facilitate an earlier return to activity.

## Case presentation

A 20 year old male presented with sudden pain in the anterior aspect of his left knee, accompanied by a loud cracking sound. The injury was sustained whilst jumping during a game of basketball. He was unable to continue playing, and weight bearing became difficult as swelling progressed. Preceding this injury, he had been diagnosed with patellofemoral pain syndrome on the basis of a 3 month history of anterior knee pain. There was no history of prior trauma or surgery to the knee. He played representative indoor netball, social basketball, squash and he trained 6 days per week. His left leg was his pivoting leg for netball.

Examination at the time of presentation revealed a moderate effusion, swelling and tenderness over the inferior half of the patella, and a decreased range of motion. Straight leg raise (SLR) was painful, but intact. The patient had a tight ITB, as assessed by the Iliotibial Band/Lateral Retinaculum test.[14]

**Figure 1:**
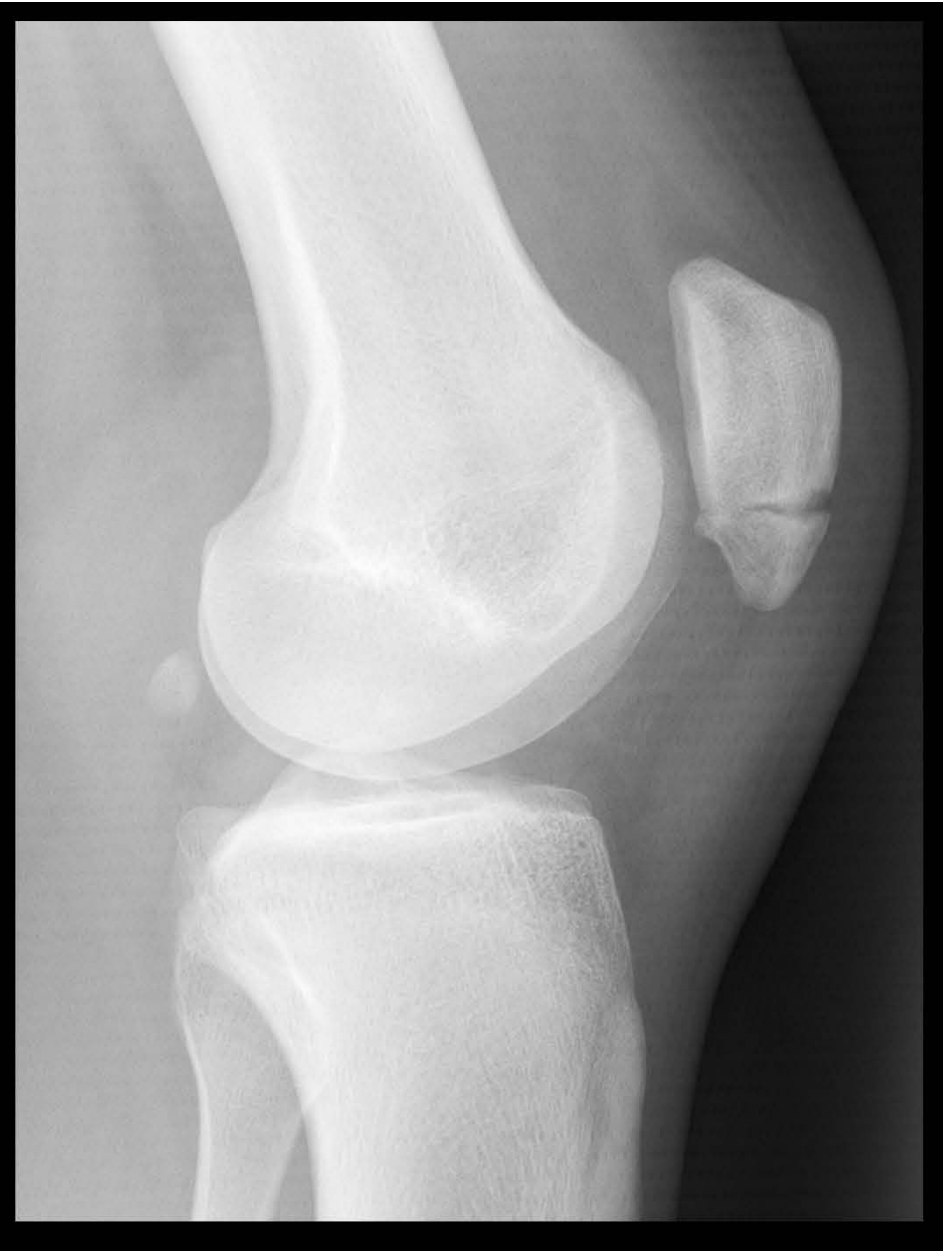
**Lateral radiograph of patient's knee showing minimally displaced transverse fracture of inferior third patella**.

**Figure 2:**
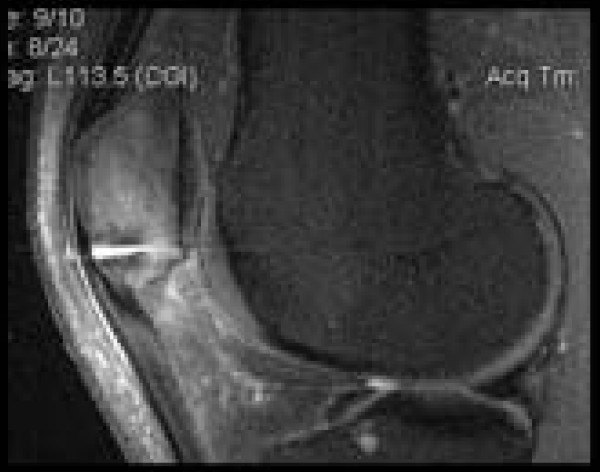
**Sagittal plane T2 MRI showing fracture, surrounding bony oedema, and sclerosis of fracture margins**.

Radiographic examination revealed a transverse fracture at the junction of the mid and distal thirds of the left patella (Fig 1). MRI scan confirmed the fracture and also revealed sclerosis at the margins of the fracture site (Fig 2).

**Figure 3:**
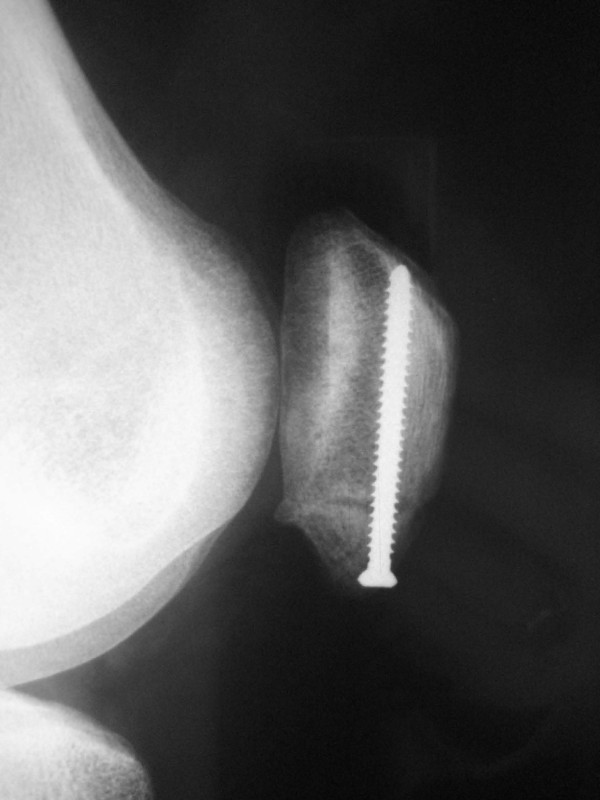
**Post-operative lateral radiograph of patella showing reduction and screw fixation of transverse patella fracture**.

The patient underwent operative treatment of the patella fracture (Fig 3). An anterior longitudinal incision was made over the patella, the fracture was exposed, and the sclerotic fracture margins were debrided. The fracture was then reduced and compression applied with a clamp. Fixation was achieved with a Synthes fully threaded 3.5 mm cortical screw. Examination under anaesthesia confirmed a tight ITB and this was subsequently released. A 2 cm transverse incision was made at the level of the superior pole of the patella, and the ITB was completely divided transversely at this level. Subsequent examination using the ITB/Lateral Retinaculum test demonstrated decreased ITB tension.

Post-operatively, the patient was allowed to weight bear as tolerated, and a Zimmer knee splint was worn for the first 2 weeks. Rehabilitation was then commenced as per the McConnell knee program,[15] modified to allow return to sport at 6 weeks. Range of motion exercises were commenced at 2 weeks post-operatively when uncomplicated wound healing was documented. Muscle strengthening and proprioception training commenced at 4 weeks. At 6 weeks he returned to unrestricted training, and 3 weeks later to unrestricted sport. The patient was able to return to his premorbid level of sport. His Lysholm knee scores were: 25 post-injury; 37 immediately post-operatively; 93 at 6 weeks; and 94 at 12 months post-operatively.

## Discussion

The first report of patellar stress fracture was in 1943 by Muller,[16] and then in the English literature by Devas, in 1960.[3] Stress fractures of the patella have since been reported in athletes, military recruits, patients with knee flexion contractures secondary to cerebral palsy,[17-19] and post-operatively following patellar surgery.[20] They account for less than 1% of all stress fractures. There have been 21 case reports of patellar stress fracture in athletes in the English literature since 1960 (Table 1).[1-13]

**Table 1:** Characteristics of previously reported cases

**Author**	**Age**	**Sex**	**Sport**	Type*	**Location**	**Displaced**	**Treatment^**	**Outcome**
Devas[3]	23	M	Running	V	Lateral	Yes	↓ activity, excision at 3 months	Full return to running
	
	28	M	Running/hockey	T	Mid 1/3	No then Yes	ORIF (sutures)	Full sports

Dickason[4]	12	M	Soccer/basketball	T	Distal 1/3	No	Cast	Full function

Iwaya[7]	12	M	Sprinting	V	Lateral	Min	↓ activity	Sport 5 months
	
	11	M	Fencing	V	Lateral	No	↓ activity	Sport 2 months
	
	10	F	Gymnastics	V	Lateral	No	↓ activity	Sport 3 months

Schranz[12]	27	M	Running	V	Lateral	Min	Excision	Not reported

Jerosch[8]	20	M	Soccer	T	Distal 1/3	Yes	ORIF (TBW)	Normal 2 yrs

Rockett[11]	20	M	Basketball	T	Distal 1/3	No	Not reported	Not reported

Teitz[13]	23	M	Skiing/sailboarding	T	Distal 1/3	Min	ORIF (TBW)	Near normal 18 months
	
	36	F	Belly dancing	T	Distal 1/3	No	Cast	Good function 6 months

Pietu[10]	16	M	Skiing/basketball	T	Distal 1/3	Min	Splint	Sport 1 yr

Garcia Mata[6]	23	M	Soccer	T	Distal 1/3	Min	Splint/Cast	Sport 3 months

Orava[9]	25	M	Volleyball	T	Distal 1/3	Min	Cast	Sport 6 months
	
	19	F	Running	V	Lateral	Min	↓ activity, excision at 5 months	Training 2 months post excision
	
	19	F	High jump	T	Distal 1/3	No	ORIF (TBW)	Sport 6 months
	
	22	M	Soccer	T	Mid 1/3	Min	Cast, ORIF, bone graft	Sports 3 yrs (↓ level)
	
	21	F	Orienteering	T	Distal 1/3	Yes	ORIF (TBW)	Sport 6 months

Brogle[1]	22	M	Basketball	T	Distal 1/3	Min	ORIF (screw + TBW)	Sport 10 months

Garcia Mata[5]	12	M	Soccer	T	Distal 1/3	Min	ORIF (screw)	Training 6 weeks

Crowther[2]	35	M	Tennis	T	Distal 1/3	No then Yes	ORIF (TBW)	Normal 10 months

*V = Vertical; T = Transverse

^ORIF = Open reduction internal fixation; TBW = Tension band wiring

Stress fractures are defined as a partial or complete fracture of bone due to an inability to withstand a non-violent stress that is applied in a rhythmic, repetitive, sub-threshold manner.[21] They may be subdivided into two types: fatigue and insufficiency. Fatigue fractures occur in bone subjected to submaximal stress loads, such as in athletes or patients with cerebral palsy.[6,22-24] Insufficiency fractures occur in previously weakened bone under physiologic stress, such as post patellar resurfacing arthroplasty or cruciate ligament reconstruction using patellar tendon.[20]

To explain the aetiology of stress fractures, two theories have been proposed: muscle fatigue, and direct muscle action. Muscle fatigue reduces its ability to absorb shock, thus allowing greater force transmission to bone, which increases local stress.[25] Conversely, muscle contraction itself creates a repetitive stress at a localised site within the bone.[26] Patellar stress fractures probably result from a combination of these mechanisms.

The classic presentation of patellar stress fracture is a young athlete with acute onset of severe anterior knee pain, often associated with a crack or pop, and inability to continue sport.[1-13] The patient has a high intensity, high frequency training program, often involving running or jumping. There is a history of gradual onset of peripatellar pain of weeks to years duration prior to the acute injury. There is usually no history of previous injury to the knee. There is localised swelling and tenderness over the inferior half of the patella, occasionally a small effusion or mildly decreased range of movement (ROM), and often a decreased ability to weight bear or straight leg raise.

The acute episode corresponds to progression from localised microfractures to complete fracture. Early diagnosis therefore minimises the need for surgery. The fracture may occur in either a transverse or vertical direction. Radiographs are sensitive initially in only one third of cases, and later in only half.[27] A skyline view is essential. MRI or bone scan will be positive early and should be used in cases where radiographs are normal.

Anatomically, there are numerous tendinous and ligamentous attachments to the patella, which act as static and dynamic stabilisers. The attachments of the extensor mechanism are important biomechanically and pathologically. The thin distal patella is enveloped by the patellar tendon over its distal third, while the thicker proximal patella is covered anteriorly by the superficial layer of the quadriceps tendon over its proximal two thirds.[5] In addition to the quadriceps and patellar tendons, lateral structures play an important role in the aetiology of patellar stress fractures. The lateral retinaculum attaches to the patella on its lateral side, and is the main lateral stabiliser. The ITB divides into the iliopatellar band (IPB) and the iliotibial tract (ITT) at the level of the knee joint.[28,29] Through these two structures, the ITB has many distal attachments, including the distal femur, lateral intermuscular septum, and Gerdy's tubercle, via the ITT; and the lateral aspect of patella and patellar tendon, via the IPB.[28,29] Both the superficial oblique and deep transverse layers of the lateral retinaculum arise mostly from the IPB.

When the knee is flexed, contraction of the quadriceps compresses the patella against the femoral condyles and trochlea. This patellofemoral compression force increases with the magnitude of quadriceps contraction and the degree of knee flexion. In 30 degrees of flexion, the quadriceps force required to maintain stance is equal to 2.1 times body weight.[30] In deep knee flexion, the patellofemoral joint reaction force is around 6–7 times body weight.[31,32]

In the flexed knee, bending stress is applied to the patella, with the anterior surface subjected to tensile forces, and the subchondral zone under compression. Finite element analysis and microradiographic studies have demonstrated that the trabecular orientation in the patella corresponds to the principal stress directions,[33,34] and that trabecular density corresponds to the magnitude of the stress.[35] Previous authors have suggested that these forces are directly responsible for the propagation of patellar stress fractures.[1,13] Transverse patellar stress fractures typically occur at the junction of the middle and distal thirds. The anatomic arrangement of the attachments of the patellar and quadriceps tendons causes a local concentration of stress, which predisposes to the development of stress fractures at this junction.

Beyond 30 degrees knee flexion, the patella engages the trochlea, and the ITB and IPB pass posterior to the lateral femoral condyle. The ITB thus exerts a posterolateral force on the patella in this range. Therefore, if the ITB is tight, the patellofemoral joint reaction force is increased.[36] This concept is supported by many biomechanical and clinical studies in the literature. Several authors have suggested that ITB tightness is a contributing factor for patellofemoral pain syndrome (PFPS),[14,15,37-41] and lateral patellar maltracking.[38,42-46] Others have suggested ITB stretching as a treatment for patellofemoral dysfunction.[42,47-49] Lateral retinacular tightness is well recognised in patients with various forms of patellofemoral dysfunction.[36,48,50-56] Puniello found a significant association between medial patellar glide and ITB flexibility in patients with PFPS.[38] He theorised that the anatomic arrangement of the ITB and lateral retinaculum provides a mechanism whereby vertical shortening of the ITB could result in horizontal shortening of the lateral retinaculum. Wu and Shih used radiographs and CT scans to assess the influence of proximal ITB release on patellar tracking, and found a significant improvement in congruence and tilt angles, which was greater than that reported for lateral retinacular release.[44]

To date, no complications directly related to release of the ITB have been reported in the literature, making the procedure relatively safe.

## Conclusion

Stress fractures of the patella are rare. The diagnosis is often missed, and radiographs may be normal. The risk of progression in undisplaced patellar stress fractures is high. A high index of suspicion and further investigation with MRI or bone scan is recommended in the athlete with anterior knee pain and localised patellar tenderness.

Non-operative management with immobilisation and observation is indicated for undisplaced fractures. Patients who require an immediate return to activity, or who have a displaced fracture, should be treated with open reduction and internal fixation. Return to sport can be expected between 6 and 12 weeks.

We recommend that ITB release should be considered as an adjunct at the time of fracture fixation. By reducing patellofemoral stress, this procedure may be of benefit in the management of patellar stress fracture.

## Consent

Written informed consent was obtained from the patient for publication of this case report and accompanying images. A copy of the written consent is available for review by the Editor-in-Chief of this journal.

## Competing interests

The authors declare that they have no competing interests.

## Authors' contributions

AK was involved in analysis and interpretation of data, review of the literature and drafting and revision of the manuscript. PB was involved in study conception, interpretation of data, and revision of the manuscript. PC was involved in acquisition of data, and revision of the manuscript. RM was involved in study conception, acquisition and interpretation of data, and revision of the manuscript. All authors read and approved the final manuscript.
